# Characterising the Genomic Landscape of Differentiation Between Annual and Perennial Rye

**DOI:** 10.1111/eva.70018

**Published:** 2024-10-25

**Authors:** Christina Waesch, Max Pfeifer, Steven Dreissig

**Affiliations:** ^1^ Institute of Agricultural and Nutritional Sciences Martin‐Luther‐University Halle‐Wittenberg Halle (Saale) Germany; ^2^ German Centre for Integrative Biodiversity Research (iDiv) Halle‐Jena‐Leipzig Leipzig Germany

**Keywords:** genomic differentiation, life‐history, recombination, rye

## Abstract

Annuality and perenniality represent two different life‐history strategies in plants, and an analysis of genomic differentiation between closely related species of different life histories bears the potential to identify the underlying targets of selection. Additionally, understanding the interactions between patterns of recombination and signatures of natural selection is a central aim in evolutionary biology, because patterns of recombination shape the evolution of genomes by affecting the efficacy of selection. Here, our aim was to characterise the landscape of genomic differentiation between weedy annual rye (*Secale cereale* L.) and wild perennial rye (*Secale strictum* C. Presl), and explore the extent to which signatures of selection are influenced by recombination rate variation. We used population‐level sequence data of annual and perennial rye to analyse population structure and their demographic history. Based on our analyses, annual and perennial rye diverged approximately 26,500 years ago (ya) from an ancestral population size of ~85,000 individuals. We analysed patterns of genetic diversity and genetic differentiation, and found highly differentiated regions located in low‐recombination regions, indicative of linked selection. Although all highly differentiated regions, as revealed by *F*_ST_‐outlier scans, were located in low‐recombining regions, not all chromosomes showed this tendency. We therefore performed a gene ontology enrichment analysis, which showed that highly differentiated regions comprise genes involved in photosynthesis. This enrichment was confirmed when *F*_ST_ outlier scans were performed separately in low‐ and intermediate‐recombining regions, but not in high‐recombining regions, suggesting that local recombination rate variation in rye affects outlier scans. Cultivated rye is an annual crop, but the introduction of perenniality may be advantageous in regions with poor soil quality or under low‐input farming. Although the resolution of our analysis is limited to a broad‐scale, knowledge about the evolutionary divergence between annual and perennial rye might support breeding efforts towards perennial rye cultivation.

## 1 Introduction

Annuality and perenniality represent fundamentally different life‐history strategies in plants. Annual plants live and reproduce in 1 year, whereas perennial plants live for at least 3 years. These different life histories are expected to influence population‐level diversity and inbreeding, with perennials experiencing higher inbreeding (Friedman 2020). In addition, perennial plants are expected to have lower effective population sizes due to longer life spans (Waples 2022). The genus *Secale* comprises three species: *S. sylvestre* Host (annual, self‐compatible, autogamous), *S. strictum* C. Presl (perennial, allogamous) and *S. cereale* L. (annual, self‐incompatible, allogamous), most of which are generally outcrossing via wind‐pollination but with different levels of self‐incompatibility (Frederiksen and Petersen 1998; Maraci, Özkan, and Bilgin 2018; Schreiber et al. 2021). Within annual *S. cereale*, there are cultivated, weedy and feral populations, whereas *S. strictum* is considered a wild perennial ancestor (Rabanus‐Wallace et al. 2021; Schreiber et al. 2022, 2021; Sun et al. 2021). While cultivated rye is annual, there are efforts to introduce perennialty into cultivated forms, which is thought to be advantageous on poor soils or in low‐input farming systems (Acharya, Mir, and Moyer 2004; Gruner and Miedaner 2021; Jaikumar et al. 2012; Reimann‐Philipp 1986). Among the *Poaceae*, there are several annual and perennial species pairs, including rice, sorghum, maize and rye (Chapman et al. 2022). In the genus *Secale*, the genomic divergence of wild annual (*S. cereale*) and wild perennial (*S. strictum*) species is not well understood. Genomic patterns of differentiation are commonly analysed through genome‐sequence or single nucleotide variant (SNV) data in divergent populations (Wolf and Ellegren 2017). While genomic regions with high genetic differentiation (*F*_ST_) might reveal ‘speciation genes’, a similar pattern might arise due to reduced genetic diversity in low‐recombining regions (i.e., linked selection) (Cutter and Payseur 2013; Wolf and Ellegren 2017). Signatures of linked selection can be inferred from levels of population differentiation (*F*_ST_), since elevated levels of differentiation can arise from both positive and purifying selection and their effects on linked neutral sites (B. Charlesworth, Morgan, and Charlesworth 1993; Chase, Vilcot, and Mugal 2024; Keinan and Reich 2010; Slotte 2014; Stephan 2019). However, elevated population differentiation could also arise from divergent selection with gene flow between populations (Guerrero and Hahn 2017; Malinsky et al. 2015). In that case, absolute sequence divergence (*d*_xy_) between populations is expected to positively correlate with levels of differentiation (*F*_ST_). Recently, patterns of divergent selection were identified in birds and plants, where local or genome‐wide patterns of *F*_ST_ and *d*_xy_ were positively correlated (Shang et al. 2023; Zhou et al. 2024). In contrast, linked selection is expected to generate a negative correlation between *F*_ST_ and *d*_xy_ (Cruickshank and Hahn 2014; Wolf and Ellegren 2017). In recent studies, genomic scans of population differentiation (*F*_ST_) were used to identify speciation islands or to characterise patterns of linked selection (Burri et al. 2015; Liang et al. 2022; Shang et al. 2023; Wang et al. 2022; Zhang et al. 2022), essentially confirming theoretical predictions regarding the relationship between recombination and patterns of linked selection (Charlesworth, Nordborg, and Charlesworth 1997). However, as signatures of selection may vary between populations and species, so may underlying recombination landscapes. Given the increasing availability of large‐scale population genetic data sets, chromosome‐scale reference genomes and recombination maps, it is now possible to characterise relationships between recombination and selection in greater detail (Marks et al. 2021; Sun et al. 2022).

Here, our aim was to characterise the landscape of genomic differentiation between wild annual (*S. cereale* L.) and wild perennial (*S. strictum* C. Presl) rye, and to test the hypothesis that differentiation between the two species was mainly influenced by the underlying recombination landscape. This was based on theoretical predictions and empirical observations on the effect the local recombination landscape on signatures of linked selection (Burri et al. 2015; Charlesworth, Nordborg, and Charlesworth 1997; Charlesworth and Wright 2001; Wang et al. 2022). To address this question, we used population‐level sequence data of wild annual rye and wild perennial rye, and analysed patterns of recombination (*ρ*), genetic differentiation (*F*_ST_) and between population absolute sequence divergences (*d*_xy_). Population structure and demographic history analysis revealed a recent split (~26,000 ya) and ongoing gene flow. We found a negative correlation between *F*_*ST*_ and *recombination rate*, and a positive correlation between *recombination rate* and *d*_xy_, suggesting that genomic differentiation between annual and perennial rye was affected by recombination rate and linked selection. At a broad‐scale, we only detect weak signatures of divergent selection (i.e., a positive correlation between *F*_ST_ and *d*_xy_). A gene ontology enrichment analysis revealed genes involved in photosynthesis in highly differentiated regions, hinting at mechanisms related to photosynthetic rate being under selection in annual and perennial rye. These insights might support breeding efforts towards perennial rye cultivation.

## 2 Materials and Methods

### 2.1 Plant Material and Genome Sequence Data

We used reduced representation genome sequencing data (genotyping‐by‐sequencing, GBS) of a *Secale* diversity panel comprising 1397 individuals of 266 accessions (Schreiber et al. 2022). Among these, we selected 158 weedy annual *S. cereale* individuals of 65 accessions and 120 wild perennial *S. strictum* individuals of 21 accessions based on available genebank passport data, prior phenotypic characterisation and prior population structure information (Schreiber et al. 2022, 2019). Variant data were retrieved from the European Variant Archive under project number PRJEB51528. Passport data of selected samples are provided in Table S1.

### 2.2 Population Structure and Demographic Modelling

The original variant matrix was filtered using VCFtools (0.1.16) (Danecek et al. 2011) to contain only biallelic single nucleotide variants (SNVs), less than 30% missing data per site, a minimum and maximum read depth per site of 10 and 100, respectively. A final variant matrix comprising 108,229 sites was used to analyse population structure via principal component analysis based on genetic co‐variance using SNPrelate (algorithm = ‘xact’, eigen. Method = ‘DSPEVX’) (Zheng et al. 2012). Estimation of individual ancestry coefficients from *K* = 2 to *K* = 4 was done using sNMF (Frichot et al. 2014). Ancestry coefficients we averaged across 20 replications using CLUMPP applying the LargeKGreedy algorithm with 500 permutations (Jakobsson and Rosenberg 2007). Demographic history and effective population size were inferred using GADMA (2.0.0) using the moments engine (ordinary differential equations) (Jouganous et al. 2017; Noskova et al. 2023). Only 47,225 neutral intergenic SNVs were used for this analysis. SNVs were annotated using SnpEff (4.0) (De Baets et al. 2012). Theta (*θ* = 4 μL) was calculated assuming a mutation rate of 3.5 × 10^−9^ (Lin, Morrell, and Clegg 2002). We used *θ* = 0.1, based on an effective sequence length (*L* = (X − Y)/X×N_seq_) ranging from 5,763,329 to 8,233,328 due to read numbers per sample ranging from 3.5 M to 5 M (100 bp reads), with *X* referring to the total number of SNVs, *Y* referring to the number of filtered SNVs and N_seq_ referring to the number of basepairs covered. For using GADMA, the site frequency spectrum (SFS) was estimated using easySFS (https://github.com/isaacovercast/easySFS) (Gutenkunst et al. 2009).

### 2.3 Gene Density, Nucleotide Diversity (*π*), Absolute Sequence Divergence (*d*_xy_) and Genetic Differentiation (*F*_ST_)

Gene density and all diversity statistics were calculated and averaged in non‐overlapping genomic windows of 10 Mb. We calculated gene density based on high‐confidence gene models in the rye ‘Lo7’ reference genome assembly v1 (Rabanus‐Wallace et al. 2021). Nucleotide diversity (*π*), absolute sequence divergence (*d*_xy_) and genetic differentiation (*F*_ST_) were calculate using the PopGenome package in R (Pfeifer et al. 2014). *F*_ST_‐values were *Z*‐transformed using the following formula: $Z\left(F_{\mathrm{ST}}\right)=\frac{F_{\mathrm{ST}}-E\left[F_{\mathrm{ST}}\right]}{\mathrm{sd}\left[F_{\mathrm{ST}}\right]}$ where $E\left[F_{\mathrm{ST}}\right]$ and $\mathrm{sd}\left[F_{\mathrm{ST}}\right]$ denote the expected value and standard deviation of *F*_ST_.

### 2.4 Recombination Landscape Analysis

We used single‐nucleotide‐polymorphism (SNP) data to estimate population recombination rates (*ρ* = *4N*_e_ × *r*) based on coalescent theory via LDhat (Auton and McVean 2007; Stumpf and McVean 2003). LDhat was run using 96 randomly selected annual or perennial individuals for 10,000,000 iterations, a block penalty of two, a population scaled mutation rate (*θ*) of 0.001, sampling every 5000 runs and the first 100,000 runs were discarded as burn‐in. A block penalty of two was chosen to reveal a greater level of detail than under a block penalty of five, and the population‐scaled mutation rate of 0.001 was chosen due to the availability of a pre‐calculated lookup table for 96 diploid unphased individuals. Different block penalties and population scaled mutation rates were tested and compared visually. While these parameters affect estimates of rho, the overall recombination landscape remains unaffected (Figure S4). *ρ* values were averaged in non‐overlapping genomic windows of 10 Mb along the chromosome. Low‐recombining regions were defined as 10 Mb windows below to 0.25 recombination rate percentile (Baker et al. 2014; Choo 1998; Fuentes et al. 2021; Schreiber et al. 2022). Recombination rate estimates are provided in Table S2. Population recombination rates (*ρ*) were correlated with available meiotic recombination rates (cM/Mb) based on a bi‐parental population in 10 Mb genomic windows (Bauer et al. 2017; Rabanus‐Wallace et al. 2021).

### 2.5 Gene Ontology Enrichment Analysis

Gene ontology (GO) enrichment analysis was done using agriGO v.2.0 (Tian et al. 2017) via singular enrichment analysis (SEA). We extracted gene IDs and GO‐terms of genes located in 10 Mb windows showing *Z*(*F*_ST_)‐values above the genome‐wide 0.95 percentile (Table S3). Additionally, 0.95 percentiles were also calculated for low‐, intermediate‐ and high‐recombining regions separately. Singular enrichment analysis was performed using gene IDs and GO‐terms of all high‐confidence genes as reference (34,433 high‐confidence genes) (Table S4). Statistical tests were conducted using Fisher's exact test and multiple‐test adjustment method after Yekutieli (significance level of 0.05), with minimum number of entries set to five. Gene enrichment ratios were calculated as the ratio of the number of significant (*p*_FDR_ < 0.05) enriched genes belonging to a GO‐term against the total number of genes belonging to the same GO‐term in the entire genome. GO‐term enrichment results are provided in Tables S5–S8.

## 3 Results and Discussion

### 3.1 Population Structure and Demographic History of Annual and Perennial Rye

**FIGURE 1:**
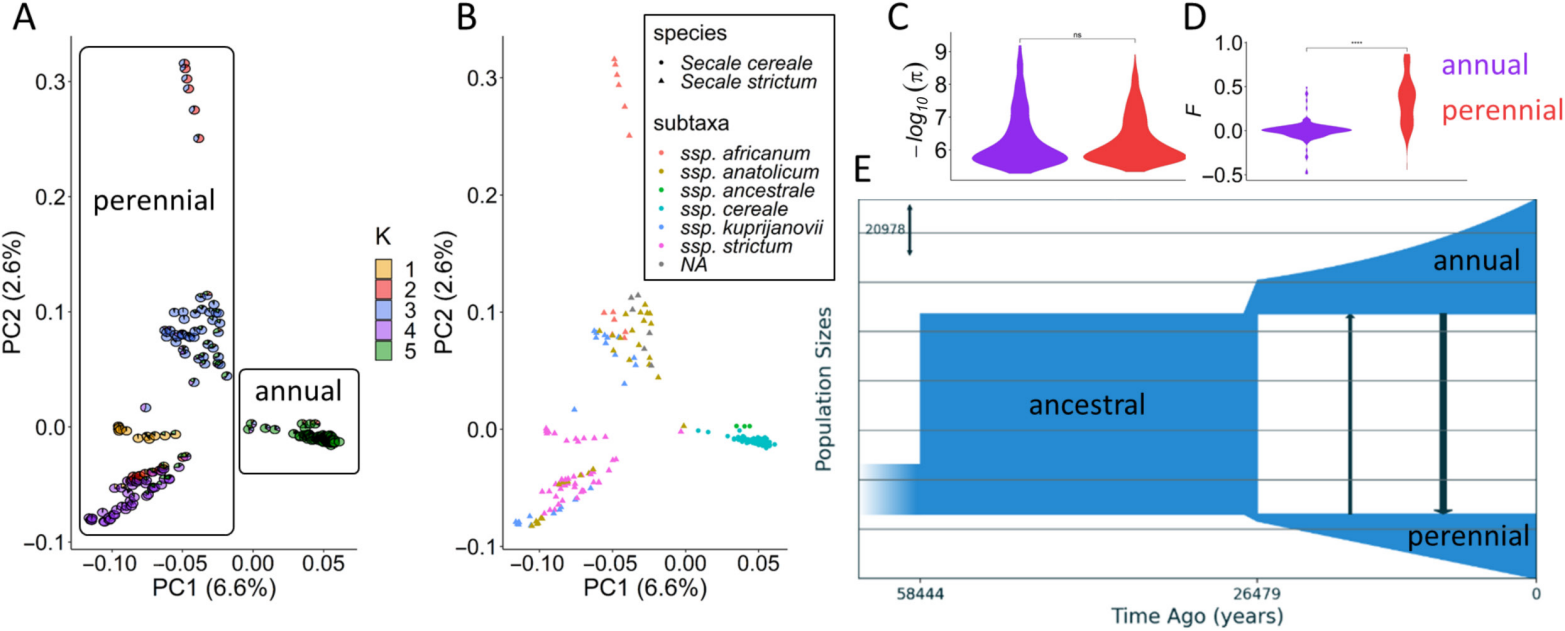
Population structure and demographic history of annual and perennial rye. (A) and (B) Principal component analysis. Annual and perennial populations are surrounded by rounded rectangles. Ancestry proportions (*K* = 5) are shown by different coloured pie charts. (C) Genome‐wide mean nucleotide diversity. (D) Inbreeding coefficient (*F*). (E) Demographic model. Gene flow between annual and perennial rye is indicated by black arrows.

Annuality and perenniality represent two fundamentally different life‐history strategies in plants. In the genus *Secale*, both annual (*S. cereale*) and perennial (*S. strictum*) species exist. Although described as separate species, previous studies showed low levels of genetic differentiation and hybrid compatibility, although with reduced fertility (Gruner and Miedaner 2021; Schreiber et al. 2019; Singh 1977; Stutz 1957). Here, we sought to characterise population structure and demographic history of annual (weedy) and perennial (wild) rye using genome sequence data of 158 and 120 individuals, respectively. A principal component analysis (PCA) based on 108,229 SNVs showed a clear separation between annual and perennial rye individuals, and four sub‐clusters among perennial rye (Figure 1A). Based on this, we estimated individual ancestry coefficients (*K* = 5), which confirmed the separation of annual and perennial rye, and further supported the sub‐clustering among perennial rye (Figure 1B). However, the overall level of genetic differentiation between annual and perennial rye was low (*F*_ST_ = 0.22) and suggestive of gene flow, which is in agreement with previous reports (Rabanus‐Wallace et al. 2021; Schreiber et al. 2019). Nucleotide diversity (*π*) did not differ significantly between annual and perennial rye, with a genome‐wide mean of *π* = 1.19 × 10^−6^ and *π* = 1.08 × 10^−6^, respectively (*p* = 0.16, Bonferroni adjusted *P*‐value, Wilcoxon rank‐sum test) (Figure 1C). Population genetic theory predicts higher levels of inbreeding in long‐lived perennials (Friedman 2020), which was also detectable in this case, with perennials showing a mean inbreeding coefficient of *F* = 0.35, and annuals a median of *F* = 0 (*p* = 4.7 × 10^−30^, Bonferroni adjusted *p*‐value, Wilcoxon rank‐sum test) (Figure 1D). Next, we inferred the demographic history of annual and perennial rye based on 47,225 neutral SNVs. While the overall phylogeny of the genus *Secale* is well characterised based on previous morphological and population genomic studies, approximate divergence times of annual and perennial rye remain less well understood, partially because of a clear lack of hybridisation barriers, incomplete linage sorting and gene flow due to wind‐pollination (Frederiksen and Petersen 1998; Li et al. 2021; Maraci, Özkan, and Bilgin 2018; Rabanus‐Wallace et al. 2021; Schreiber et al. 2019, 2021; Sun et al. 2021). Here, the most likely demographic model revealed an estimated ancestral population size of *N*_*a*_ ~ 85,000 individuals approximately 58,000 years ago (ya). Annual and perennial rye diverged approximately 26,000 ya, and exhibit reduced contemporary effective population sizes of *N*_*e*_ ~ 49,000 for annual rye and *N*_*e*_ ~ 27,000 for perennial rye (Figure 1D). The rates of inferred gene flow were higher from annual to perennial rye than vice versa, with a per‐generation migration rate of 2.3 × 10^−5^ and 9.4 × 10^−6^, respectively. These observations meet theoretical expectations, where perennial populations are predicted to have lower effective population sizes due to longer generation times (Friedman 2020; Waples 2022).

### 3.2 Genomic Landscape of Differentiation Between Annual and Perennial Rye

In order to explore the genomic landscape of differentiation between annual and perennial rye, we analysed patterns of genetic differentiation (*F*_ST_), between‐population sequence divergence (*d*_xy_) and population recombination rates (*ρ*). Patterns of genetic differentiation throughout the genome are influenced by an interplay between natural selection and the underlying recombination landscape. Theory and empirical data predict a positive correlation between recombination rate and patterns of genetic diversity due to linked selection, that is, the reduction of genetic diversity at neutral sites linked to the actual targets of selection (Brian Charlesworth and Jensen 2021). This is expected to drive genetic differentiation between populations in low‐recombining regions (Shang et al. 2023; Slotte 2014; Wolf and Ellegren 2017).

**FIGURE 2:**
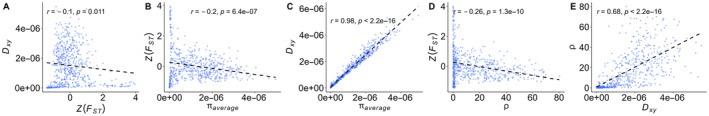
Genome‐wide correlation between genetic differentiation, sequence divergence, recombination rate and nucleotide diversity. (A) Pearson correlation (*r*) between absolute sequence divergence (*d*_xy_) and genetic differentiation (*Z*(*F*_ST_)). (B) Pearson correlation (*r*) between genetic differentiation (*Z*(*F*_ST_)) and average nucleotide diversity (*π*_average_). (C) Pearson correlation (*r*) between nucleotide diversity (*π*_average_) and absolute sequence divergence (*d*_xy_). (D) Pearson correlation (*r*) between genetic differentiation (*Z*(*F*_ST_)) and population recombination rate (*ρ*, estimated in annual rye). (E) Pearson correlation (*r*) between population recombination rate (*ρ*, estimated in annual rye) and absolute sequence divergence (*d*_xy_).

Here, we estimated *F*_ST_, *d*_xy_ and *ρ* in non‐overlapping genomic windows of 10 Mb. Across all genomic windows, we observed a positive correlation between absolute sequence divergence (*d*_xy_) and recombination rate (*ρ*) of *r* = 0.68 (*p* < 2.2 × 10^−16^), a negative correlation between genetic differentiation (*F*_ST_) and recombination rate (*ρ*) of *r* = −0.26 (*p* = 1.3 × 10^−10^), and a negative correlation between *d*_XY_ and *F*_ST_ of *r* = −0.1 (*p* = 0.01) (Figure 2). The correlations between recombination rate and *F*_ST_ and *d*_xy_ were confirmed using a reference genetic map, with *r* of −0.2 and 0.42, respectively, (*p* < 1.1 × 10^−6^). These patterns suggest that genomic differentiation between annual and perennial rye is shaped by linked selection at broad‐scale.

**FIGURE 3:**
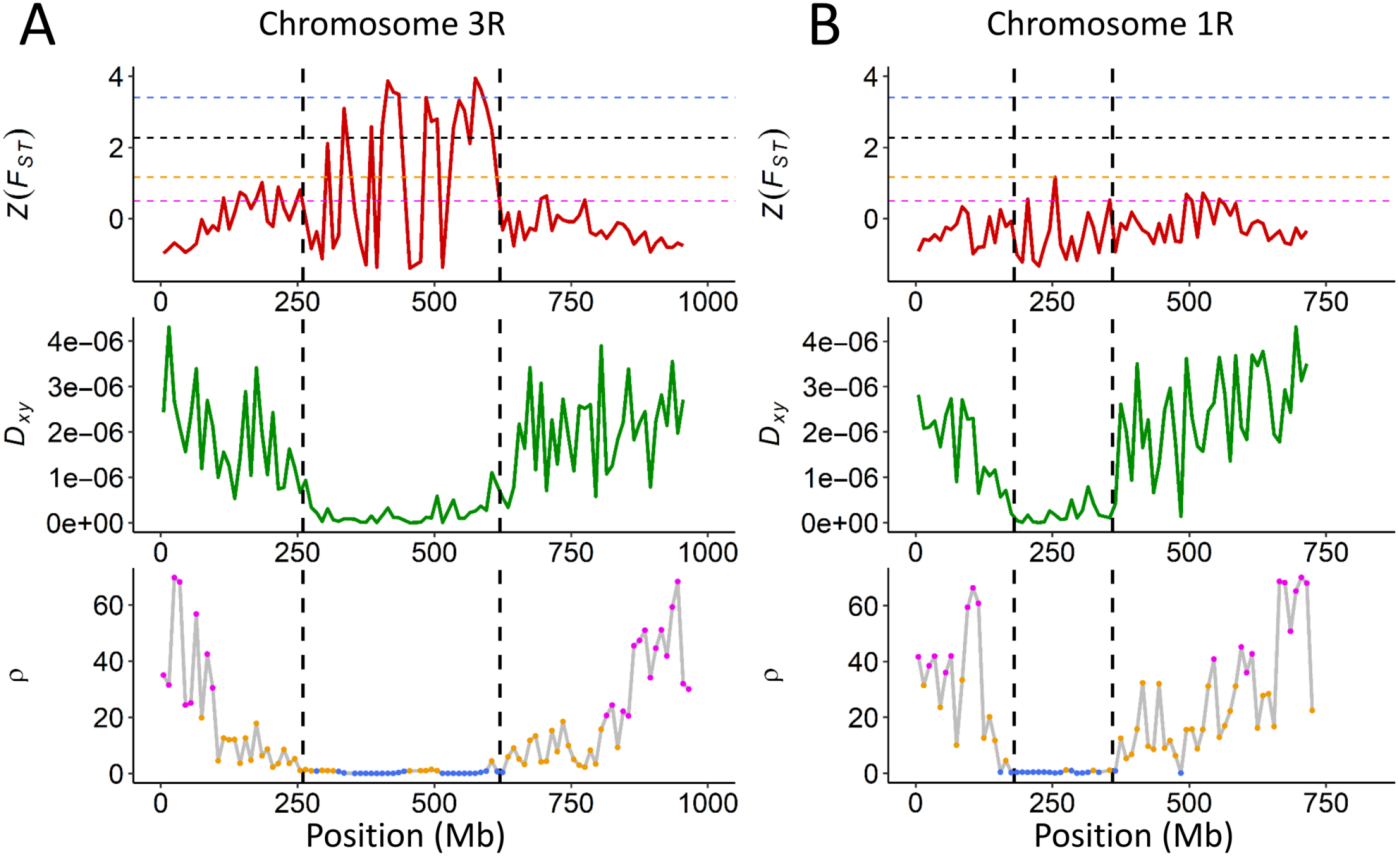
Heterogeneous differentiation landscapes. Presence (A) and absence (B) of signatures of linked selection on chromosomes 3R and 1R, respectively. Dashed vertical lines delineate the low‐recombining region of each chromosome. Dashed horizontal lines correspond to *F*_ST_‐outlier scans based on a genome‐wide threshold (black), low‐recombining regions (blue), intermediate‐recombining regions (orange) and high‐recombining regions (magenta).

The recombination landscapes of annual and perennial rye are characterised by large low‐recombining regions, spanning 25%–50% of each chromosome and comprising an average of 7%–25% of all genes (Figure 3A,B). We performed *F*_ST_ outlier scans in order to identify genomic windows of significant differentiation (> 0.95 percentile), which identified 31 of 600 windows (5.2% of the genome), among which all were located in low‐recombining regions (Figure 3, Figure S1). This implies that linked selection is driving the differentiation between annual and perennial rye. However, not all chromosomes showed significantly differentiated windows in low‐recombining regions (i.e., chromosomes 1R and 7R), and the genome‐wide correlations between *F*_ST_ and *ρ*, and *d*_Xy_ and *F*_ST_, were low (*r* = −0.2, *r* = −0.1, respectively), suggesting that patterns of differentiation were not solely caused by linked selection. Since *F*_ST_ outlier scans were shown to be influenced by recombination rate variation throughout the genome (Booker, Yeaman, and Whitlock 2020), we performed outlier scans in low‐, intermediate‐ and high‐recombining regions independently. This way, we found varying numbers of significantly differentiated bins, with nine in low‐recombining regions (5.9%), 59 in intermediate‐recombining regions (19.5%) and 144 in high‐recombining regions (75%). Interestingly, we observed a positive correlation between *F*_ST_ and *d*_XY_ on chromosome 1R, which might be caused by divergent selection, but no *F*_ST_ outliers above the genome‐wide threshold were found (Figure 3B, Figure S1, *r* = 0.23, *p* = 0.049).

### 3.3 Genomic Context of Highly Differentiation Regions

**FIGURE 4:**
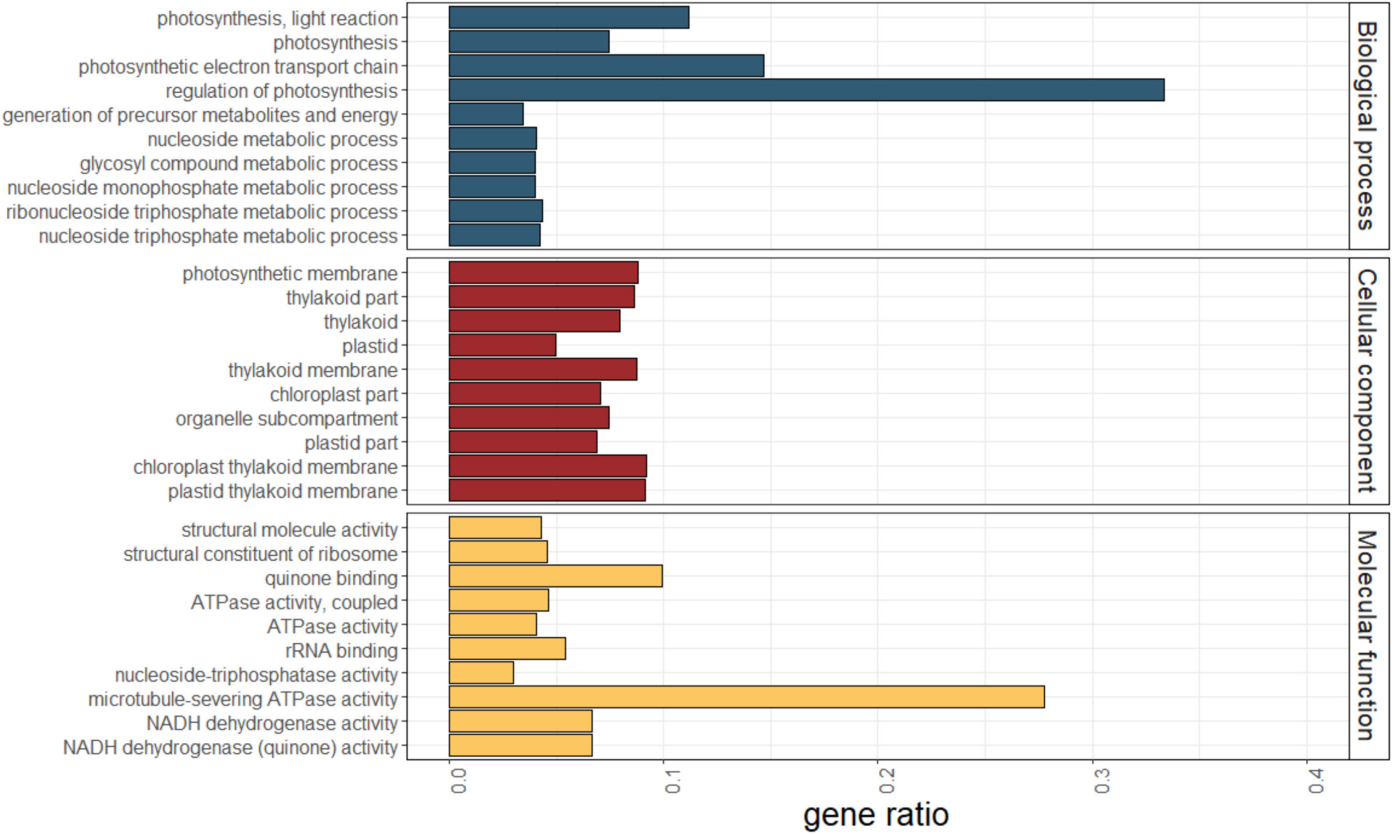
Gene ontology enrichment in significantly differentiated regions. Gene ontology terms are separated into ‘Biological process’, ‘Cellular component’ and ‘Molecular function’. Within each class, we included the top 10 terms with the highest gene enrichment ratios, sorted by *p*_FDR_‐value in ascending order. A full list of all GO‐terms is available in Table S5.

While significantly differentiated genomic regions often overlap with low‐recombining regions, as was shown in a wide range of species (Burri et al. 2015; Cutter and Payseur 2013; Lehnert et al. 2020; Liang et al. 2022; Wang et al. 2016, 2022; Wolf and Ellegren 2017; Zhang et al. 2022), it is intriguing to ask which genes and functional classes are located therein, and are therefore potential targets of selection. To address this question, we performed a gene ontology (GO) enrichment analysis based on genes in significantly differentiated regions. Under a genome‐wide significance threshold, a total of 426 annotated high‐confidence (HC) genes were located in 31 significantly differentiation windows, which amounts to 5.1% of the genome. Among these, a total of 163 GO‐terms were significantly enriched (*p* < 0.05), with 81 belonging to GO class ‘biological process’, 44 to ‘cellular component’ and 38 to ‘molecular function’. Within each class, most significantly enriched genes were involved in photosynthesis (Figure 4).

Since *F*_ST_‐outlier scans were shown to be influenced by recombination rate variation (Booker, Yeaman, and Whitlock 2020), we also performed this analysis separately for low‐, intermediate‐ and high‐recombining regions. In both low‐ and intermediate‐recombining regions, we found significant differentiation in genes related to photosynthesis (Figure S2, Tables S6 and S7). In high‐recombining regions, however, significantly differentiated windows harbour genes related to response to biotic and abiotic stress (Figure S2, Table S8).

Long‐lived perennial plants maintain vegetative growth after flowering (Albani and Coupland 2010; Rohde and Bhalerao 2007; Scofield and Schultz 2006), which might explain why these genes are significantly differentiated between annual and perennial rye. Interestingly, evolutionary theory suggests perennial plants to have lower instantaneous photosynthetic rates due to a trade‐off between resource accumulation and seed yield versus risk of mechanical damage and stress tolerance (Ehleringer 1995; Garnier 1992; Grime 1977), while empirical work comparing annual and perennial cereal crops showed that perennial forms have 10%–50% higher photosynthetic rates (Nikhil S. Jaikumar, Snapp, and Sharkey 2013). Either way, differences in photosynthetic rates between annual and perennial plants might explain why genes involved in these processes are highly differentiated between annual and perennial rye. On the contrary, since the available reduced representation genome sequencing data only allowed us to analyse patterns of differentiation at a broad scale, that is, in genomic windows of 1%–2% of the physical length of the rye genome, these genes may be linked to other targets of selection in low‐recombining regions. Previous work by (Gruner and Miedaner 2021) revealed genomic loci associated with perenniality via genome‐wide association scans, but none of these loci overlap with the significantly differentiated genomic regions identified here. Genomic differentiation between annual *S. cereale* and perennial *S. strictum* was also analysed by (Rabanus‐Wallace et al. 2021), who observed strong differentiation along the entire low‐recombining region on chromosome 4R, indicative of linked selection. In our work, low‐recombining regions of chromosomes 3R and 6R showed strongest differentiation, and only a smaller region of approximately 75 Mb on chromosome 4R showed significant differentiation. Since Rabanus‐Wallace et al. (2021) used domesticated‐like annual rye, whereas our work utilises weedy annual rye, this apparent difference might be caused by different selective pressures, such as human selection throughout crop domestication versus natural selection in wild populations.

In conclusion, we show an unprecedented analysis of the genomic landscape of differentiation between weedy annual and wild perennial rye. On a broad‐scale genome‐wide level, genetic differentiation (*F*_ST_) is negatively correlated with the population recombination rate (*ρ*), and absolute sequence divergence (*d*_xy_) is positively correlated with *ρ*, revealing widespread signatures of linked selection. While highly differentiated regions mostly overlapped with low‐recombining regions of the chromosome, not all chromosomes were equally affected (i.e., chromosome 1R and 5R). A gene ontology enrichment analysis revealed that highly differentiated genomic windows are mostly comprising genes related to photosynthesis, suggesting that the regulation of photosynthesis might play an important role in perennials versus annuals. This may be expected, since perennials maintain vegetative structures after flowering, which are expected to have increased photosynthesis over generative structures (Friedman 2020). A limitation of our study is the resolution at which selection signals can be inferred. Given that reduced representation sequencing data (genotyping‐by‐sequencing, GBS) only covers a small fraction of the genome, our analyses can only reveal broad‐scale patterns in genomic windows of 1%–2% of the physical length of the genome. Further work using high‐coverage whole‐genome‐sequencing data will be required to identify the actual targets of selection in annual and perennial rye.

## Conflicts of Interest

The authors declare no conflicts of interest.

## Supporting information


**Figure S1.** (A) Correlation between genetic differentiation and recombination rate based on a reference genetic map. (B) Correlation between recombination rate and absolute sequence divergence.

**Figure S2.** Genomic differentiation landscape. (A) Recombination rate based on reference genetic map in cM/Mb (Bauer et al. 2017). (B) Population recombination rate estimated in annual rye. (C) Genetic differentiation between annual and perennial rye. (D) Absolute sequence divergence between annual and perennial rye. (E) Correlation between population recombination rate (*ρ*, estimated in annual rye) and absolute sequence divergence (*d*_XY_). (F) Correlation between population recombination rate (*ρ*, estimated in annual rye) and genetic differentiation (Z(*F*_ST_)). (G) Correlation between absolute sequence divergence (*d*_XY_) and genetic differentiation (Z(*F*_ST_)). Dashed vertical lines delineate the low‐recombining region of each chromosome. Dashed horizontal lines correspond to *F*_ST_‐outlier scans based on a genome‐wide threshold (black), low‐recombining regions (blue), intermediate‐recombining regions (orange) and high‐recombining regions (magenta).

**Figure S3.** GO‐term enrichment performed based on *F*_ST_‐outlier scans performed separately in low‐, intermediate‐ and high‐recombining regions.

**Figure S4.** Comparison of LD‐hat parameters.

**Table S1** Passport data of rye samples used in the present data.
**Table S2**. Estimates of population recombination rates in annual and perennial rye.
**Table S3**. ID and ontology terms of genes located in 10 Mb windows showing *F*_ST_‐values above the 0.95 percentile.
**Table S4**. ID and ontology terms of all high‐confidence genes in the rye genome (Lo7 v1 genome assembly).
**Table S5**. GO‐term enrichment results under genome‐wide threshold.
**Table S6**. GO‐term enrichment results under low‐recombining threshold.
**Table S7**. GO‐term enrichment results under intermediate‐recombining threshold.
**Table S8**. GO‐term enrichment results under high‐recombining threshold.
**Table S9**. Summary of estimates of diversity statistics in genomic windows used for correlation analyses.

## Data Availability

Rye accessions used in this study are listed in Table S1. Variant data are available at the European Nucleotide Archive under project number PRJEB51528. Data used for correlation analyses are provided in Table S9.
