# Supplementary figures and images for: Characterising the Genomic Landscape of Differentiation Between Annual and Perennial Rye
Source: Evol Appl. 2024 Oct 25;17(10):e70018. doi: 10.1111/eva.70018 (PMC11511776; doi:10.1111/eva.70018)

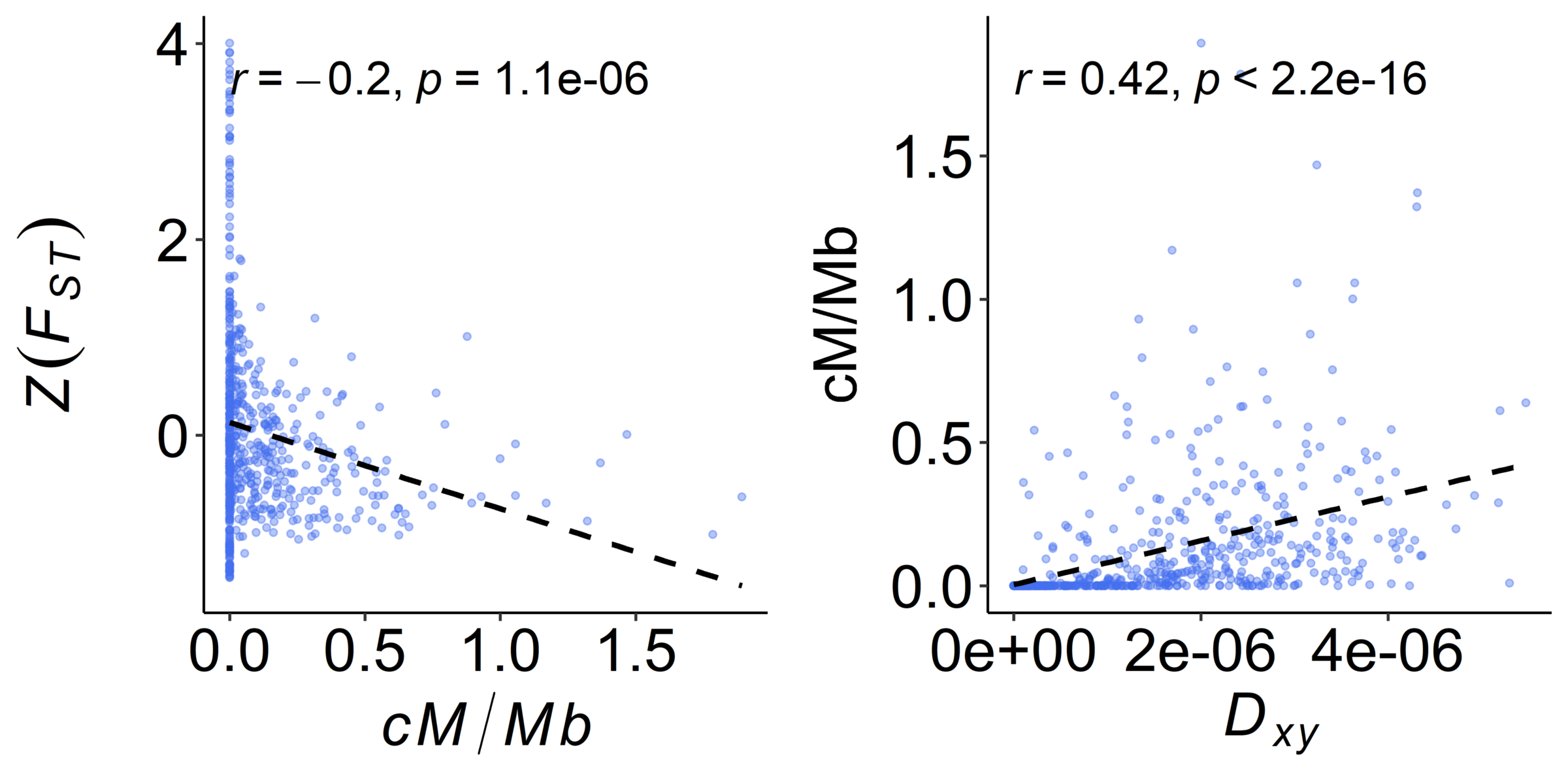

Supplement: Supplementary file 1 — Figure S1. (A) Correlation between genetic differentiation and recombination rate based on a reference genetic map. (B) Correlation between recombination rate and absolute sequence divergence. [file EVA-17-e70018-s003.tif]

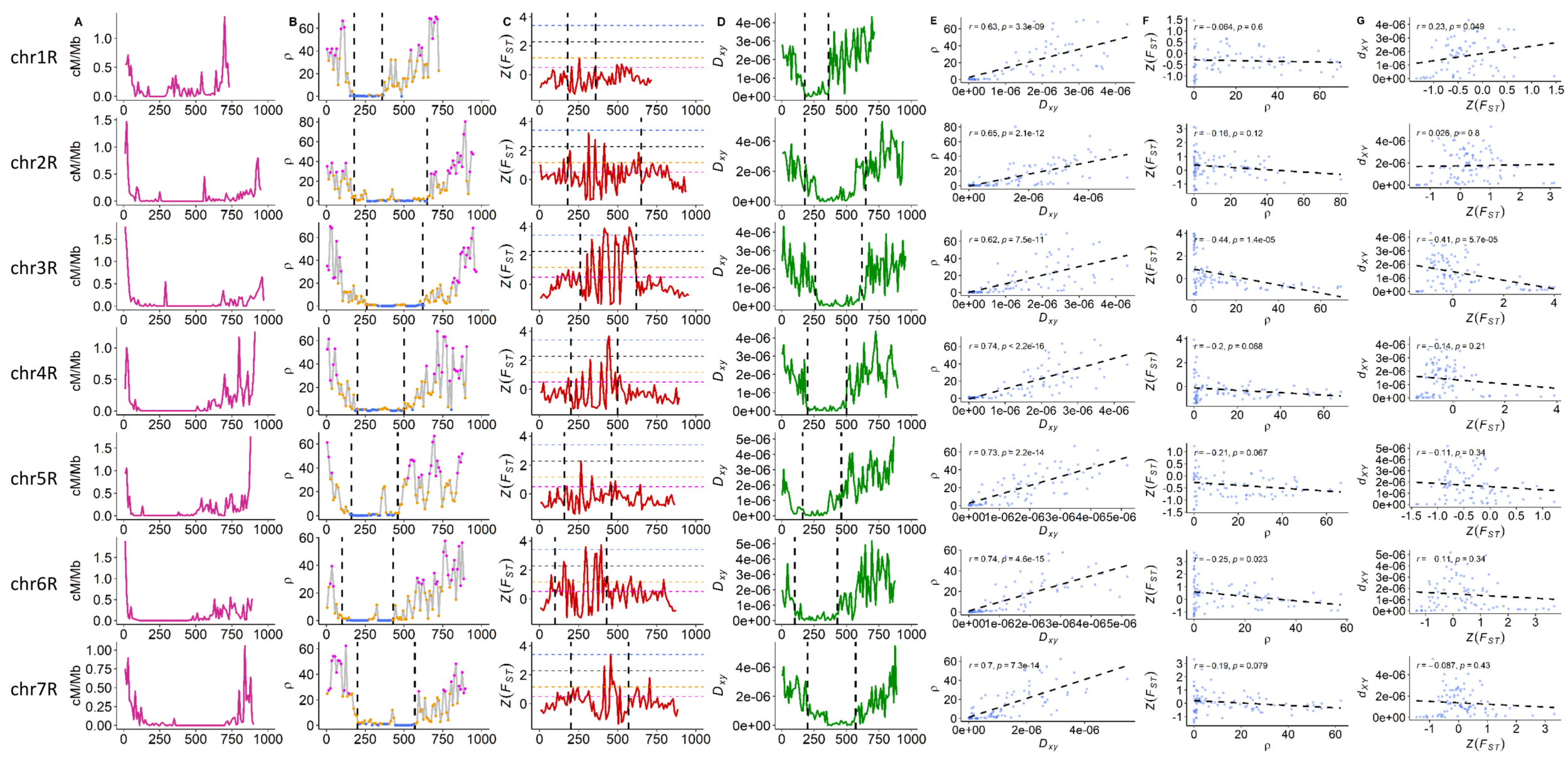

Supplement: Supplementary file 2 — Figure S2. Genomic differentiation landscape. (A) Recombination rate based on reference genetic map in cM/Mb (Bauer et al. 2017). (B) Population recombination rate estimated in annual rye. (C) Genetic differentiation between annual and perennial rye. (D) Absolute sequence divergence between annual and perennial rye. (E) Correlation between population recombination rate (ρ, estimated in annual rye) and absolute sequence divergence (d XY). (F) Correlation between population recombination rate (ρ, estimated in annual rye) and genetic differentiation (Z(F ST)). (G) Correlation between absolute sequence divergence (d XY) and genetic differentiation (Z(F ST)). Dashed vertical lines delineate the low‐recombining region of each chromosome. Dashed horizontal lines correspond to F ST‐outlier scans based on a genome‐wide threshold (black), low‐recombining regions (blue), intermediate‐recombining regions (orange) and high‐recombining regions (magenta). [file EVA-17-e70018-s005.tif]

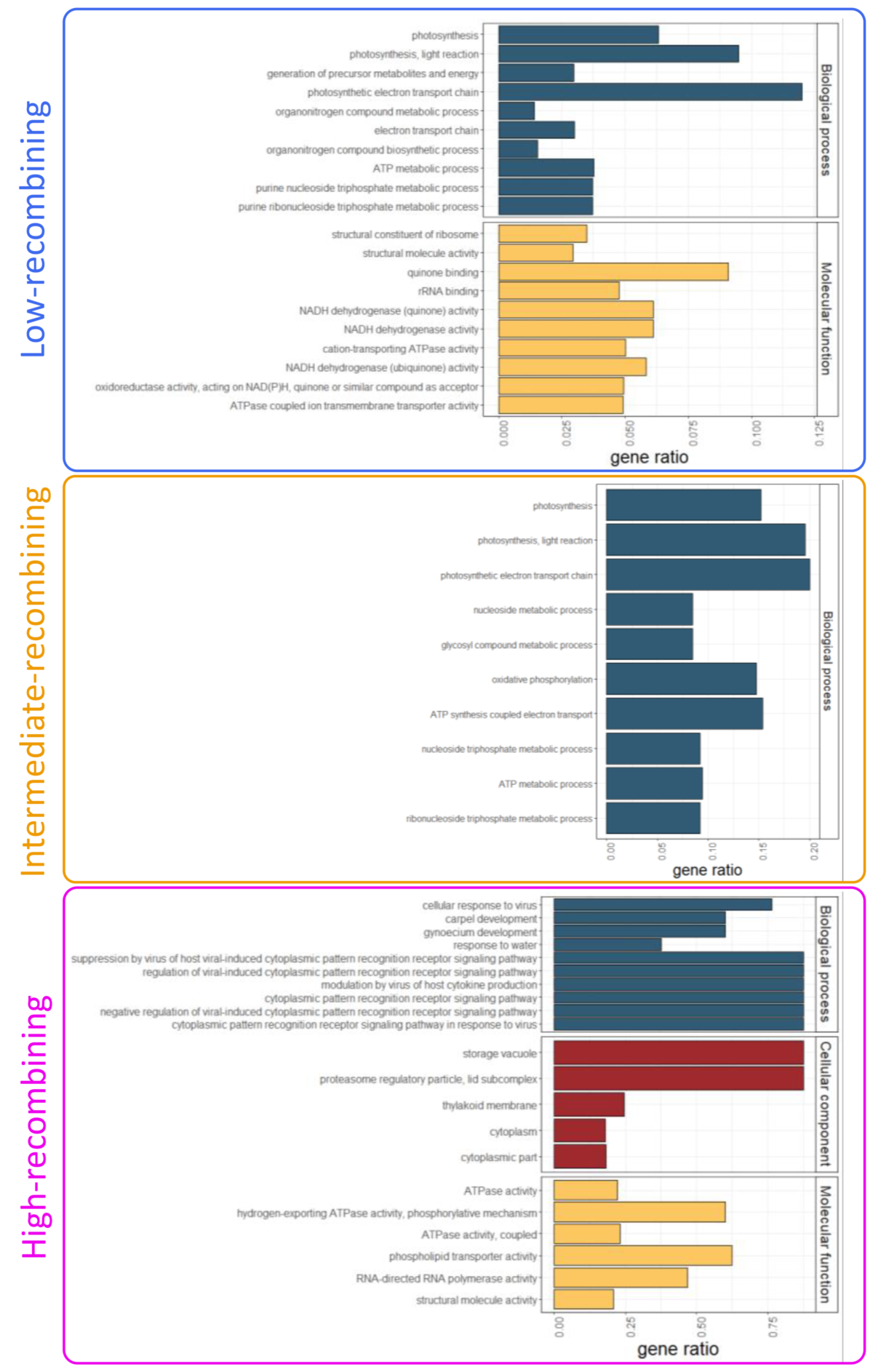

Supplement: Supplementary file 3 — Figure S3. GO‐term enrichment performed based on F ST‐outlier scans performed separately in low‐, intermediate‐ and high‐recombining regions. [file EVA-17-e70018-s001.tif]

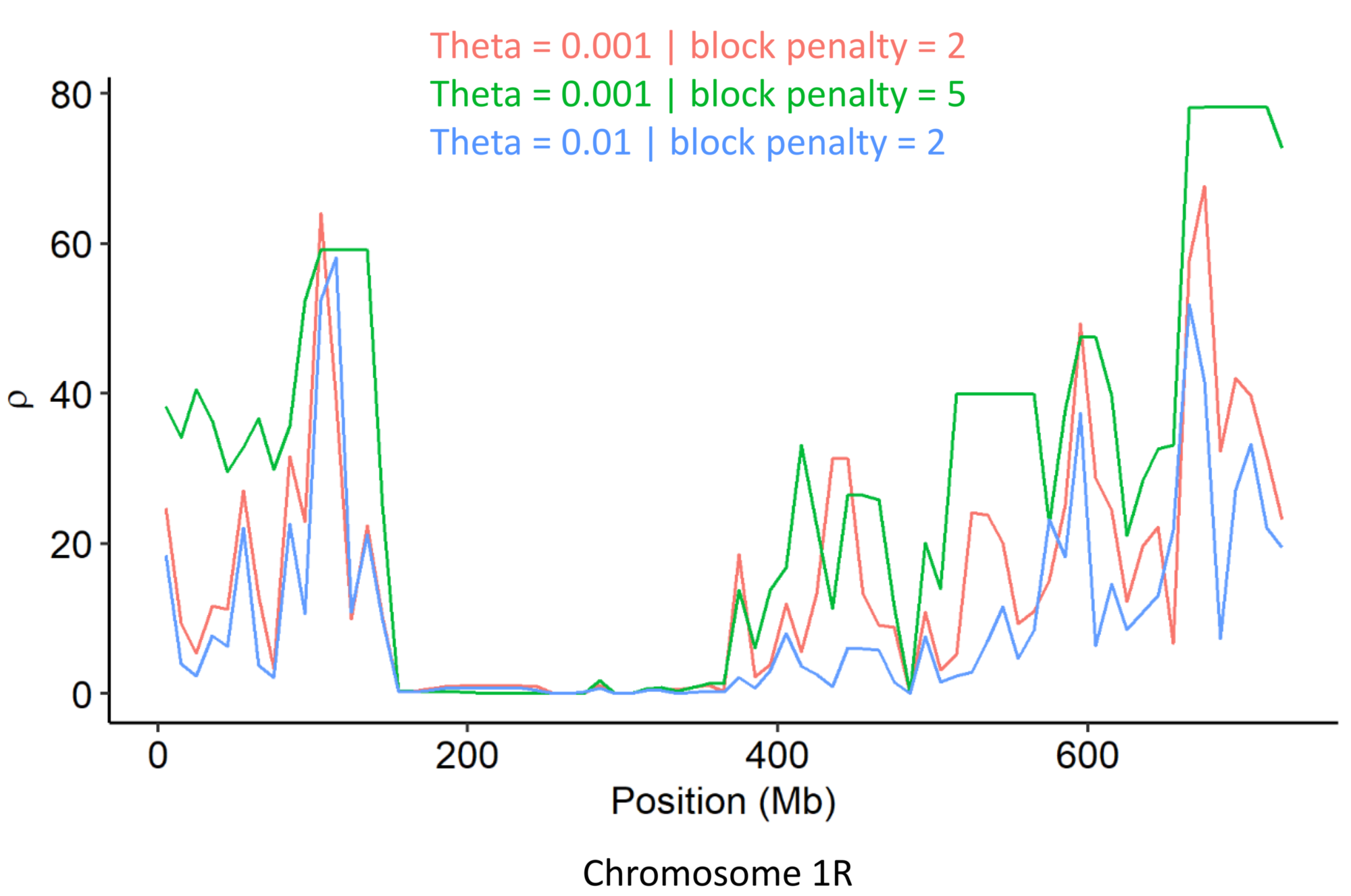

Supplement: Supplementary file 4 — Figure S4. Comparison of LD‐hat parameters. [file EVA-17-e70018-s004.tif]
